# P-272. Comparing HIV Recent Diagnoses Among Sexual Risk Strata in African Nations: Insights from the Subnational HIV Estimates in Priority Populations (SHIPP) Tool

**DOI:** 10.1093/ofid/ofaf695.493

**Published:** 2026-01-11

**Authors:** Jacob R Miller, Kathryn Risher

**Affiliations:** Pennsylvania State University, Hershey, PA; Pennsylvania State University, Hershey, PA

## Abstract

**Background:**

An estimated 67% of those living with HIV reside in Sub-Saharan Africa. With limited resources for public health interventions, targeting high-risk populations for preventative health interventions is crucial. The Subnational HIV Estimates in Priority Populations (SHIPP) tool was developed to assist in defining high risk populations for public health intervention design. The purpose of the present analysis was to examine HIV new diagnoses across SHIPP-defined sexual risk categories (those with no sexual partners, sexually active with a single cohabitating partner, and sexually active with non-regular partners) in 13 African nations with historically high HIV incidence.

**Methods:**

Using Population-Based HIV Impact Assessment (PHIA) data from 95,423 individuals, logistic regression was used to compare the likelihood of testing positive for HIV within the previous year between sexual risk categories.

**Results:**

The odds of testing positive for HIV between sexual risk categories varied widely between nations and between sexes. In general, females sexually active with a single partner were more likely to have a new positive HIV test compared to sexually inactive females (OR 2.74 [1.48, 5.07] in Cameroon, 2.61 [1.14, 5.94] in Rwanda), though those with non-regular partners showed elevated risk in Lesotho (OR 2.84 [1.94, 4.12]). There were few nations that showed differences in HIV new diagnoses among males, though males in Malawi and Tanzania were significantly less likely to test positive if they had non-regular partners compared to none (0.52 [0.28, 0.97], 0.37 [0.15, 0.86]).

**Conclusion:**

These results highlight the complex transmission dynamics that exist in high-burden nations, as well as differences in transmission dynamics between sexes. Countries with significant differences in HIV new diagnoses between sexual risk categories would benefit from targeted interventions for high-risk groups, especially for women with non-regular sexual partners. Others may benefit from broader prevention strategies or additional research to identify high-risk groups. These findings can inform resource allocation for targeted intervention planning and underscore the value of risk stratification tools in intervention planning.

**Disclosures:**

All Authors: No reported disclosures

